# Extreme Rapid Eye Movement Rebound Exceeding Three Hours During Continuous Positive Airway Pressure Titration Despite High-Dose Antidepressant Therapy: A Case Report

**DOI:** 10.7759/cureus.97988

**Published:** 2025-11-27

**Authors:** Rafeek Kandy, Ulhas Jadhav, Babaji Ghewade, Irfan Shafiq, Khaled Saleh

**Affiliations:** 1 Respiratory and Allergy Institute, Cleveland Clinic Abu Dhabi, Abu Dhabi, ARE; 2 Respiratory Therapy, Datta Meghe Institute of Higher Education & Research, Wardha, IND; 3 Respiratory Medicine, Jawaharlal Nehru Medical College, Datta Meghe Institute of Higher Education & Research, Wardha, IND

**Keywords:** antidepressants, cpap titration, obstructive sleep apnea, rem rebound, sleep homeostasis

## Abstract

Rapid eye movement (REM) rebound following the initiation of continuous positive airway pressure (CPAP) is commonly observed, with episodes exceeding two hours being rare. We report a 38-year-old male with severe obstructive sleep apnea (OSA) (apnea-hypopnea index (AHI) = 74.2/hour) receiving fluoxetine 80 mg daily and trazodone 100 mg, who had a 206.5-minute continuous REM sleep episode during CPAP titration, followed by 66.5 minutes of stage N3 sleep. This duration exceeds previously reported continuous REM episodes during titration. This case demonstrates that severe OSA-related sleep fragmentation can overcome potent pharmacological REM suppression when effective treatment is initiated.

## Introduction

Rapid eye movement (REM) rebound refers to a compensatory increase in REM sleep duration and percentage following periods of suppression or deprivation [1,2]. It is well established after withdrawal of REM-suppressing antidepressants, sleep deprivation, and after initiation of continuous positive airway pressure (CPAP) in obstructive sleep apnea (OSA). In OSA, REM rebound on the titration night is often defined as a 20% rise in percentage REM (%REM) compared to the diagnostic study. This threshold was first derived by Brillante et al. (2012) [3] and later popularized in clinical teaching by Palen et al. (2016) [4] and other reviews [1]. By the same derivation, slow-wave sleep (stage N3) rebound is defined as 40% or greater [3]. Prevalence of REM rebound varies with the threshold used. Approximately 23% of patients experience rebound with a threshold of 20% or greater, and up to 46% with a lower threshold of 6% [1].

OSA disrupts normal sleep architecture through repetitive respiratory events that fragment sleep and particularly affect REM sleep, reflecting a lower arousal threshold relative to airway reopening during REM. This chronic disruption creates a physiological sleep debt that becomes apparent as rebound once positive airway pressure (PAP) restores ventilation. REM rebound is associated with higher oxygen desaturation index (ODI), higher arousal index, lower mean oxygen saturation (SpO₂), lower baseline %REM, higher Epworth Sleepiness Scale (ESS), and younger age. N3 rebound is associated with younger age, longer total apnea-hypopnea duration, shorter baseline N3 duration, lower SpO₂ nadir, and lower %REM [2].

Selective serotonin reuptake inhibitors (SSRIs) like fluoxetine commonly increase REM latency and decrease total REM sleep, and dose-dependent REM effects have been reported for both SSRIs and tricyclic antidepressants (TCAs) [5,6]. REM rebound is well documented following antidepressant withdrawal [6]. When CPAP effectively restores normal ventilation, REM rebound is commonly observed, reflecting recovery from accumulated sleep debt. However, concurrent antidepressants can affect net REM expression under CPAP, as most inhibit REM, but bupropion can have unpredictable effects [4,6].

Previous case reports have documented extreme REM rebound episodes. Lo Bue et al. (2014) reported REM sleep comprising up to 72% of total sleep time in the context of antidepressant withdrawal [7]. To our knowledge, the longest continuous REM episode during CPAP titration reported to date is 140 minutes [4], and another report describes 113 minutes [8]. We present a case demonstrating a substantially longer continuous REM episode occurring despite ongoing high-dose REM-suppressing medication therapy.

## Case presentation

A 38-year-old physically active male with a medical history of major depressive disorder, chronic insomnia, and attention deficit hyperactivity disorder presented for sleep evaluation. He was experiencing persistent fatigue for several months, which he initially attributed to his psychiatric medications. However, he also reported loud snoring and occasional choking sensations while sleeping on his back. These symptoms prompted him to consult his primary care physician, who subsequently referred him to our sleep clinic for evaluation of possible OSA.

Upon evaluation at the sleep clinic, the patient described excessive daytime sleepiness, unrefreshing sleep, witnessed apneas reported by his bed partner, early morning awakenings at 3-4 am, and significant impairment in daytime functioning affecting his work performance. He reported a typical bedtime of 8-9 pm with a sleep latency of approximately 30 minutes. He denied regular napping, alcohol consumption, or tobacco use, but consumed four cups of tea daily. Physical examination revealed a body mass index (BMI) of 32.5 kg/m². His STOP-BANG score was 5, and Epworth Sleepiness Scale score was 14. Other physical examination was unremarkable, with no evidence of upper airway abnormalities, thyromegaly, or cardiopulmonary compromise.

The patient had a complex antidepressant history beginning in 2018 with combination therapy, including citalopram, venlafaxine, vortioxetine, and bupropion. For two years preceding the study, he was maintained on trazodone 100 mg at bedtime and fluoxetine 80 mg daily, with lithium 400 mg added five months prior to the clinic evaluation. He also received eszopiclone 3 mg at bedtime. All medications except eszopiclone were continued on the sleep study night.

A split-night polysomnogram was performed following standard protocols. The baseline diagnostic portion lasted 182.5 minutes with a total sleep time of 139 minutes, yielding a sleep efficiency of 76.2%. Sleep architecture demonstrated a complete absence of both REM and N3 sleep stages, with N2 sleep comprising 90.6% of total sleep time and N1 sleep accounting for 9.4%. The apnea-hypopnea index (AHI) was 74.2 events per hour, with exclusively obstructive respiratory events. The supine AHI was elevated compared to lateral positioning (84.2 vs. 63.6 events/hour). Oxygen saturation reached a nadir of 50%, and the arousal index was 69.1 events per hour.

**Figure 1:**
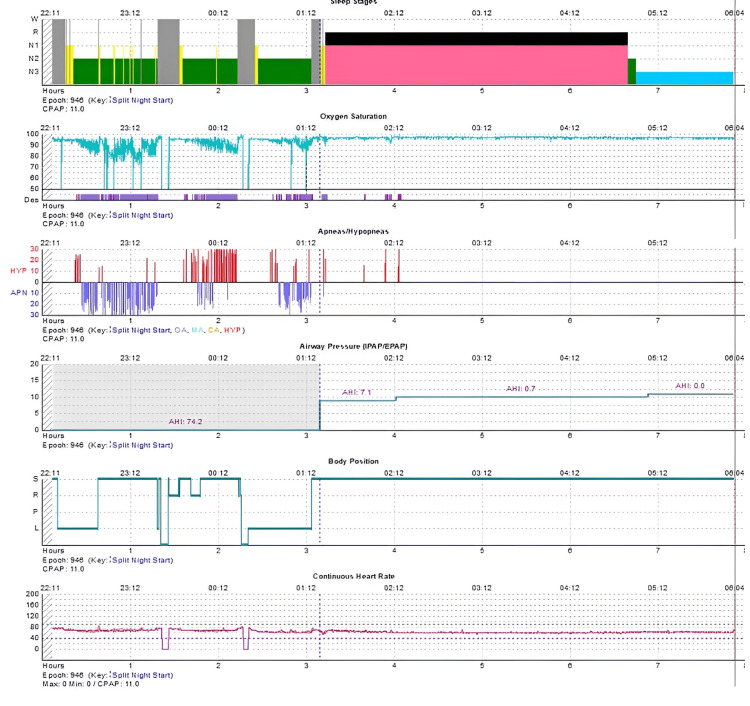
Hypnogram of CPAP titration (split-night). Baseline diagnostic period (0-3 hours) shows no REM sleep. During titration, the patient entered REM within three minutes and remained in continuous REM for 206.5 minutes, immediately followed by sustained N3 until study termination. CPAP = continuous positive airway pressure; REM = rapid eye movement sleep.

CPAP titration was initiated at 1:21 am following the diagnostic period. The patient transitioned into REM sleep within three minutes of CPAP initiation and remained in continuous REM sleep for 206.5 minutes, representing 73.5% of the total titration sleep time. This was immediately followed by N3 sleep lasting 66.5 minutes (23.7% of titration sleep time). The final therapeutic CPAP pressure was 11 centimeters of water (cmH₂O). Post-CPAP parameters included an AHI of 1.71 events per hour, an arousal index of 1.3 events per hour, and an oxygen saturation nadir of 89% (Table 1 and Figure 1).

**Table 1:** Polysomnographic parameters: baseline diagnostic vs. CPAP titration vs. entire night. Recording time, TST, sleep efficiency, stage distribution (N1, N2, N3, REM), latencies, WASO, AHI (total/REM/NREM/supine/side), oxygenation (lowest and average SpO₂), arousal indices (overall, PLM-related), awakenings, and heart rate metrics. CPAP = continuous positive airway pressure; TST = total sleep time; WASO = wake after sleep onset; AHI = apnea–hypopnea index; REM = rapid eye movement sleep; NREM = non-rapid eye movement sleep; SpO₂ = arterial oxygen saturation; PLM = periodic limb movements.

Parameter	Baseline	CPAP	Entire night
Recording time	182.5	283	465.5
Total sleep time	139	281	420
Sleep efficiency	76.16%	99.29%	90.32%
N1 duration (%)	9.40%	0.90%	3.70%
N2 duration (%)	90.60%	2.00%	31.30%
N3 duration (%)	0.00%	23.70%	15.80%
REM duration (%)	0.00%	73.50%	49.20%
N1 latency	9	1	9
REM latency	0	3	177
Sleep onset latency	9	1	9
Wake after sleep onset	34.5	1	36.5
Total AHI	74.24	1.71	38
REM AHI	NA	1.74	0.87
NREM AHI	74.24	1.61	37.9
Supine AHI	84.17	1.71	42.6
Side AHI	63.58	0	31.8
Lowest SpO2	50%	89%	50%
Average SpO2	92%	97%	95%
Arousal index	69.1	1.3	23.7
Number of awakenings	7	1	9
PLM index	10.8	10.7	10.7
PLM-arousal index	1.3	0.4	0.4
Heart rate (average)	68 bpm	63 bpm	64 bpm
Heart rate (range)	60-85 bpm	54-73 bpm	54-85 bpm

## Discussion

To the best of our knowledge, this case documented the longest continuous REM episode during CPAP titration reported to date at 206.5 minutes, exceeding previously reported durations during PAP titration. Palen et al. (2016) [4] described a 140-minute uninterrupted REM during titration, with 83% of titration sleep and 60% of total study sleep. Another report documented 113 minutes of continuous REM [8]. Notably, our patient experienced extreme REM rebound despite ongoing high-dose REM-suppressing medications (fluoxetine 80 mg daily, trazodone 100 mg, and lithium 400 mg), whereas Palen et al. (2016) [4] reported that their patient had discontinued sertraline two days before the study while continuing bupropion (which can have variable effects on REM). Pavirala and Goyal (2022) [8] stated that medication effects were ruled out in their case, with no specific mention of concurrent REM-affecting medications. This distinction emphasizes the remarkable nature of our observation, as severe OSA-related sleep fragmentation was able to overcome potent ongoing pharmacological REM suppression once effective ventilation was restored.

Other extreme cases of REM rebound differed in context, as Lo Bue et al. (2014) [7] reported around 72% of total sleep time (TST) as REM during paroxetine withdrawal but not as a single continuous sleep, and Zeidan et al. reported around 200 minutes of total REM (around 40% TST) due to a pontine lesion [9]. Notably, most previously reported extremes occurred with antidepressant withdrawal, whereas our patient remained on therapeutic doses during the study.

The patient's long-term exposure to REM-suppressing agents, particularly high-dose fluoxetine and trazodone, would ordinarily be expected to suppress REM expression, making the observed rebound remarkable. SSRIs such as fluoxetine consistently lengthen REM latency and reduce total REM; these effects are dose-dependent in both healthy volunteers and depressed patients [6]. With trazodone, the dominant effect is increased slow-wave sleep, while REM effects are variable across studies (reduced or unchanged) [10]. The discontinuation of eszopiclone likely had minimal impact, as Z-drugs have relatively modest effects on REM sleep architecture [11].

The presence of extreme REM rebound despite this pharmacological background suggests that chronic, severe sleep debt may override potent REM-suppressing effects when effective ventilation is restored. In our case, the complete absence of baseline REM and severe OSA (AHI = 74.2/hr) provided the preconditions for marked rebound once respiratory events were eliminated by CPAP.

The observed transition from prolonged REM sleep directly to sustained N3 sleep represents an interesting clinical phenomenon requiring further investigation. Following the 206.5-minute REM episode, the patient transitioned directly into 66.5 minutes of sustained N3 sleep, with minimal intervening N1/N2 epochs. While the patient was receiving trazodone (known to increase slow-wave sleep (SWS) in healthy subjects [10]), the relationship between this medication effect and the timing of the N3 episode following REM exhaustion remains unclear and requires further investigation.

We conducted methodological verification for the potential for confounding factors. The "Prozac eyes" phenomenon, characterized by fluoxetine-induced eye movements during non-rapid eye movement (NREM) sleep, required careful exclusion to ensure accurate REM identification [12]. Polysomnographic analysis confirmed genuine REM characteristics throughout the episode, including mixed-frequency, low-amplitude electroencephalogram (EEG), sustained submental atonia, and typical rapid eye movements. Additionally, care was taken to distinguish true N3 sleep from potential slow-wave artifacts that can mimic slow-wave sleep.

Several factors likely contributed to this extreme rebound response. Severe OSA with marked fragmentation and absent baseline REM suggests a significant reduction of restorative stages, with rebound expected once ventilation normalizes. The chronic nature of symptoms is consistent with accumulated sleep debt. Additionally, younger age has been associated with larger REM rebound magnitudes in titration cohorts [2,13]. Immediate and effective resolution of respiratory events during CPAP titration created optimal conditions for expression of rebound.

This case has several clinical implications. Extreme REM rebound can occur despite concurrent REM-suppressing medications, suggesting extended monitoring during titration may be warranted in select patients with severe OSA receiving antidepressant therapy. Second, greater REM rebound at titration has been associated with better early CPAP adherence, which may facilitate counseling and follow-up planning [13]. Finally, while the observed transition pattern requires further study, it may inform expectations about sleep recovery during initial CPAP treatment in patients with severe OSA and concurrent antidepressant therapy.

## Conclusions

To the best of our knowledge, this 206.5-minute continuous REM episode during PAP titration represents the longest reported duration despite ongoing high-dose REM-suppressing antidepressant therapy. This case supports that severe OSA-related sleep fragmentation can override pharmacological REM suppression once effective ventilation is restored by CPAP. While this represents a single observation and cannot be generalized, it has potential clinical implications. It may help clinicians anticipate marked REM rebound during initial CPAP titration in patients with severe OSA, even those receiving REM-suppressing medications, and allow sufficient opportunity to document REM sleep when feasible.
